# Artificial Food Color Additives and Child Behavior: Weiss Responds

**DOI:** 10.1289/ehp.1104409R

**Published:** 2012-01-01

**Authors:** Bernard Weiss

**Affiliations:** Department of Environmental Medicine, University of Rochester, School of Medicine and Dentistry, Rochester, New York, E-mail: bernard_weiss@urmc.rochester.edu

The Food and Drug Administration’s (FDA) response to my commentary (Weiss 2012) reflects the wide gulf between how the FDA translates “weight of evidence” into regulatory policy for artificial food colors (AFCs) and how it is translated into meaningful action on behalf of public protection.

The FDA essentially took the position that for a study to be considered as evidence of adverse effects, it must be totally free of uncertainties. The study by McCann et al. (2007) played a large role is provoking the FDA review, but for that study, like almost any epidemiological study, it would be difficult to meet that absolute criterion. It is why *Environmental Health Perspectives (EHP)* publishes so many such studies addressing the same question (e.g., air pollution). But isn’t it fair to ask whether any of the negative AFC studies meet that criterion?

In their critique, the FDA faults McCann et al. (2007) because they characterized “… a treatment effect as adverse when it may, in fact, fall within the normal range of childhood behavior.” This is an issue discussed over and over again in the pages of *EHP*. Take the example in my commentary (Weiss 2012), modeled on numerous publications in the lead literature (e.g., Lanphear et al. 2005): If developmental exposure to low levels of lead reduces a population IQ (intelligence quotient) by 3 points (3%), from, say, 100 to 97, it is taken as evidence of a major adverse effect. Both scores, of course, fall within the normal range. The same criticism is used by the FDA to dismiss the effect size calculations; that is, the altered behavioral activity seen in published data lies “… in the range of normal activity for children.”

The FDA finds the study by McCann et al. (2007) lacking because the authors relied mainly on parental observations. A high proportion of child development research, in fact, enlists parents as observers; hundreds of validated inventories and questionnaires are based on parent ratings. They are the observers, of course, who see the most extensive samples of the child’s behavior, especially with younger children. This is the reason I chose parental observations for my own food color study of young children (Weiss et al. 1980) and why we relied on parent ratings for our study of how phthalates mold play behavior in preschool children (Swan et al. 2010).

It is difficult to grasp the FDA argument that AFCs do not possess “inherent” neurotoxic properties but may provoke neurotoxicity in susceptible subpopulations. Neurotoxicity is neurotoxicity.

The FDA does acknowledge that AFCs may be associated with adverse behavioral outcomes in some (unknown proportion of) susceptible children. As I note in my commentary (Weiss 2012), such a conclusion would prompt decisive action by the U.S. Environmental Protection Agency. Why not the FDA?

I was pleased to hear that the FDA noted the need for further research. My question remains: What parent or institutional review board (IRB) would be convinced that such research is without significant risk, given what we already know? If IRBs would hesitate, shouldn’t that prompt the FDA to at least require warning labels on foods containing AFCs that are consumed mainly by children?

Finally, the FDA policy reflects a point of view that is endemic in federal regulatory policy toward potentially toxic chemicals. Namely, a chemical is innocent until proven guilty. Many environmental health researchers believe the proposition needs to be reversed. Some advocate adoption of the precautionary principle. Perhaps, if the FDA had required neurotoxicity testing, especially in young children, before allowing AFCs and other additives to be marketed, we would not be having this debate at all. Harvey Wiley, who became the FDA’s first commissioner, recruited his legendary “Poison Squad” volunteers for precisely this purpose. That was in 1902.
